# Limb Ischemic Conditioning Improved Cognitive Deficits via eNOS-Dependent Augmentation of Angiogenesis after Chronic Cerebral Hypoperfusion in Rats

**DOI:** 10.14336/AD.2017.1106

**Published:** 2018-10-01

**Authors:** Changhong Ren, Ning Li, Sijie Li, Rongrong Han, Qingjian Huang, Jiangnan Hu, Kunlin Jin, Xunming Ji

**Affiliations:** ^1^Institute of Hypoxia Medicine, Xuanwu Hospital, Capital Medical University, Beijing 100053, China; ^2^Department of Pharmacology and Neuroscience, University of North Texas Health Science Center, TX Texas 76107, USA.; ^3^Center of Stroke, Beijing Institute for Brain Disorder, Beijing 100069, China.; ^4^Beijing Key Laboratory of Hypoxia Translational Medicine, Beijing 100053, China; ^5^Department of Neurobiology, Capital Medical University, Beijing 10069, China

**Keywords:** limb ischemic conditioning, chronic cerebral hypoperfusion, angiogenesis, eNOS, NO

## Abstract

Intracranial and extracranial arterial stenosis, the primary cause of chronic cerebral hypoperfusion (CCH), is a critical reason for the pathogenesis of vascular dementia and Alzheimer’s disease characterized by cognitive impairments. Our previous study demonstrated that limb remote ischemic conditioning (LRIC) improved cerebral perfusion in intracranial arterial stenosis patients. The current study aimed to test whether LRIC promotes angiogenesis and increases phosphorylated endothelial nitric oxide synthase (p-eNOS) activity in CCH rat model. Adult male Sprague-Dawley rats were randomly assigned to three different groups: sham group, bilateral carotid artery occlusion (2VO) group and 2VO+LRIC group. Cerebral Blood Flow (CBF) was measured with laser speckle contrast imager at 4 weeks. Cognitive testing was performed at four and six weeks after 2VO surgery. We demonstrated that LRIC treatment increased cerebral perfusion and improved the CCH induced spatial learning and memory impairment. Immunohistochemistry confirmed that LRIC prevented cell death in the CA1 region, and increased the number of vessels and angiogenesis in the hippocampus after 2VO. Western blot analysis shows that LRIC therapy significantly increased p-eNOS expression in the hippocampus when compared with 2VO rats. Moreover, eNOS inhibitor reduced the effect of LRIC on angiogenesis in the hippocampus and spatial learning and memory function. Our data suggested that LRIC promoted angiogenesis, which is mediated, in part, by eNOS/NO.

Cerebrovascular stenosis resulting from arteriosclerosis induces failure of the cerebral circulation. Although chronic cerebral hypoperfusion (CCH) does not influence acute neural cell death [1], CCH is a critical reason for the pathogenesis of vascular dementia (VD) and Alzheimer’s disease (AD), which is characterized by cognitive impairment [2]. CCH mimics the pathological initiation of VD by permanent, bilateral occlusion of the common carotid arteries in rats [3]. Previous studies have revealed that CCH leads to cognitive deficits and neuronal loss in the hippocampus [3]. Furthermore, several studies have suggested that an association exists between 2VO-induced cognitive impairment and hippocampal damage [3]. To date, successful drug treatment has not been identified to prevent CCH induced cognitive deficits [4]. An ideal restorative approach for the prevention of tissue death after cerebral ischemia should promote vascular perfusion in the ischemic region.

Ischemic neovascularization involves three major processes: angiogenesis, arteriogenesis (collateralization), as well as post-natal vasculogenesis [5, 6]. Angiogenesis, the process of new capillary growth through sprouting from existing blood vessels, could be interpreted as a natural defense mechanism helping to restore oxygen and nutrient supply to the ischemic brain tissue [6, 7]. Besides, neuroblasts have been found to be concentrated around blood vessels following cerebral ischemia-induced injury [8]. Therefore, therapeutic angiogenesis and subsequent improvement of cerebral blood flow is an important strategy to treat and/or prevent ischemic disease [9]. So far, many approaches to angiogenesis therapy in small animal and large animal models have been reported, including drug and non-drug therapy [7]. Higher microvessel density in the ischemic border correlates with longer survival in stroke patients [10]. Thus, therapeutic enhancement of angiogenesis may provide a new treatment strategy for cerebral ischemia patients [9].

Recent studies have shown a new technique termed limb remote ischemic conditioning (LRIC), an intrinsic process whereby repeated short episodes of double-sided hind limb non-lethal ischemia/reperfusion is neuroprotective after global and focal ischemic stroke [11, 12]. Our previous study showed that repetitive bilateral arm ischemic preconditioning reduced the incidence of stroke recurrence and improved cerebral perfusion in intracranial arterial stenosis patients. However, there is limited understanding of the underlying mechanisms [13]. Recently, Esposito et al. reported that ischemic conditioning increased angiogenesis after focal cerebral ischemia [14]. However, any direct evidence that repetitive LRIC induces angiogenesis in CCH has not been postulated.

Endothelial nitric oxide synthase (eNOS) has a crucial role in the regulation of systemic blood pressure, vascular tone, vascular remodeling, and angiogenesis [5]. A study by Murohara et al. showed impaired vascular remodeling during hind limb ischemia in eNOS knockout mice [15]. Furthermore, there was a marked increase in collateral vessel formation and subsequent recovery of blood flow in the ischemic limb of eNOS transgenic mice [16]. Therefore, given the central role of eNOS signaling in the modulation of angiogenesis, we asked whether eNOS signaling might be involved in LRIC-mediated neuroprotection.

In this study, we examined the effects of LRIC on angiogenesis and eNOS signaling in a rat model of chronic cerebral hypoperfusion.

## MATERIALS AND METHODS

### Animals

All animal experiments were approved by Animal Care and Use Committee of Xuanwu Hospital, Capital Medical University, China, and conducted according to the National Institutes of Health guidelines. Thirty adult male Sprague-Dawley rats (220-260g weight) were purchased from Vital River Laboratories, Beijing, China, and maintained on a 12-hour light/dark cycle with unlimited access to food and water.

### Chronic cerebral hypoperfusion model

CCH model was induced using the double carotid artery or 2-vessel occlusion (2VO) model as described previously [25, 26]. Briefly, rats were anesthetized with 4.0% enflurane and then maintained on 1.5-2.0% enoflurane in 70% N_2_O and 30% O_2_ using a small-animal anesthesia system. Through a midline cervical incision, the bilateral common carotid arteries were carefully separated from the cervical sympathetic and vagal nerves, and then one of the arteries was doubly ligated with silk sutures. After ten minutes, the other carotid artery was also ligated. The incision was closed eventually. During the operation, rectal temperature was monitored and maintained at 37±0.5 °C with a heating blanket. The rats in sham group underwent a similar surgery except that the carotid arteries were not occluded.

### Limb remote ischemic conditioning

Rats were randomly assigned to three groups: sham-operated group, 2VO group, and 2VO + LRIC group. Each group included ten animals. LRIC was initiated at three days after the hypoperfusion model by occluding blood flow to the hind limbs bilaterally while under anesthesia, once a day for 28 days. Hind limb occlusion was accomplished by tightening a tourniquet (8 mm) around the upper thigh for three cycles. For each cycle, the occlusion and release phase lasted 10 minutes, respectively. The rats in the sham and 2VO groups were only under anesthesia as the 2VO + LRIC group.

### *Adminis*tration of antago*nist of NOS*

Vehicle or L-NG-nitroarginine methyl ester (L-NAME; 50?mg/kg per day; Sigma-Aldrich, St. Louis, MO), an antagonist of NOS was intraperitoneally injected from day seven after surgery lasting for five weeks.

### Cerebral Blood Flow(CBF) by Laser Speckle Contrast Imager (LSCI)

High-resolution LSCI (PSI system, Perimed Inc.) was used to image cerebral perfusion and record CBF at day 14 after 2VO as previously described [17]. Briefly, the anesthetized rats were placed in a stereotaxic apparatus. The regions of interest (ROIs), each with a 2.5 mm × 5 mm area that was centered at 2.5 mm posterior and 2.5 mm lateral to the bregma over the left cortex. The skull was thinned gently using a high-speed dental drill (SDE-H37L, Marathon, Korea) until the vessels were visible. Perfusion images were acquired at 23 fps (exposure time T = 5 ms) using PSI system (Perimed, Stockholm, Sweden) with a laser diode (780 nm; Dolphin BioTech Ltd., China). One blood flow image was generated by averaging the numbers obtained from 20 consecutive raw speckle images. The CBF in a region of interest was obtained by using a dedicated PIM Soft program (Perimed Inc.).

### Morris water maze

The spatial learning and memory ability of rat was evaluated by Morris water maze (MWM, DMS-2, Beijing, China) on day 21 and day 35 after 2VO surgery, respectively. Morris water maze was used to test cognitive function [1,2]. The water maze used in our study was a flat black galvanized metal tank that was 210 cm in diameter and equipped with a platform 1-2 cm below the surface of the water. The rats were trained for 5 consecutive days, followed by the probe trial on day 6 with the original platform removed. The rats were trained four times per day (120 sec/trial) and were let down in four random places (N, W, SE, NW) in the pool. If the animals failed to locate the platform within 120 seconds, they were put on the platform and stayed for 20 seconds before the next swim trial. The latency was recorded as 120 seconds. If the animals reached the platform within 120 seconds, they were immediately removed from the platform. On day 6, a spatial probe trial was conducted with the original platform removed to evaluate memory retention. The animals were let down diagonally from the platform. The cumulative time spent in target quadrant where the platform was located was recorded during a period of 60s. The results were presented as the time spent (time percentage) in the target quadrant.

### Histology and immunohistochemistry analysis

Each group of rats (N = 5 per group) were anesthetized and decapitated at six weeks after surgery. Brains were removed, and 10-µm coronal sections were prepared and stained with cresyl violet. Cell numbers were measured by a blinded observer. Immunostaining was performed on brain sections as previously described [18]. The primary antibodies were mouse monoclonal anti-NeuN (1:300, Invitrogen, USA), rat anti-ki67 (1:100, Abcam, Cambridge, UK). FITC conjugated *Lycopersicon esculentum* (Tomato) Lectin (1:300, Vector Lab, USA), which react with rat endothelial cell antigen were used for vessel staining. Slides were mounted using proLongGold antifade reagent with DAPI (Molecular Probes, USA). Fluorescence signals were detected with Nikon *Ti Eclipse* Epi-fl Illuminator (Nikon, Japan). Ki67^+^ and Lectin^+^ double-labeled cells in the hippocampus were counted in three 5-μm paraffin coronal sections per animal by an observer blinded to the experimental groups. Five rats were used for each group.

### Western blot analysis

Western blot analysis was used to assess protein expression in the hippocampus. Protein (40 µg) was electrophoresed on 10% SDS-polyacrylamide gels and then transferred to a polyvinylidene difluoride membrane (Millipore Corporation, USA). The antibodies dilutions were 1:1000 for GAP-43 and MAP-2 (Santa Cruz, CA, USA), 1:2000 for CD31 (Abcam, Cambridge, UK), eNOS and phosphorylated eNOS antibody (Cell Signaling, Boston, USA). The specific reaction was visualized using a chemiluminescent substrate (GE Healthcare, UK). GAPDH was used to verify equal loading (1:3000, Sigma-Aldrich, Missouri, USA). The optical density of protein was measured using Image-Pro Plus software 5.0 (Rockville, MD, USA) according to the manufacturer’s instructions.

### Statistical analysis

Data from the percentage of time spent in the target quadrant in MWM and field EPSP recordings in LTP experiments were expressed as mean ± S.E.M.% of baseline fEPSP amplitude and slope. Other data were expressed as Mean ± SEM. Escape latencies in MWM and date from LTP recording were compared using repeated measure ANOVA. Other data were analyzed using one-way ANOVA. The analyses were performed using SPSS 16.0 software. The significance level was set at *P* < 0.05.

## RESULTS

### LRIC treatment increased cerebral perfusion

**Figure 1.**
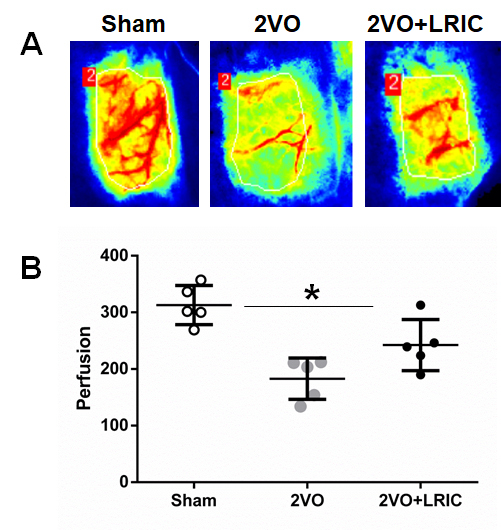
Detection of cerebral blood flow changes by laser specle contrast imager **A)** Laser speckle images taken from representative rats in sham, 2VO, and 2VO+LRIC groups. **B)** Absolute value of cerebral perfusion in three groups.

Cerebral perfusion was measured using laser speckle. Fig. 1A and B shows that the perfusion decreased significantly on day 14 after 2VO surgery. In comparison to 2VO group, there was a significant increase in CBF in the 2VO+LRIC treatment group (*P*<0.05) (Fig. 1A and B).

### LRIC treatment improved the chronic cerebral hypoperfusion induced spatial learning and memory impairment

**Figure 2.**
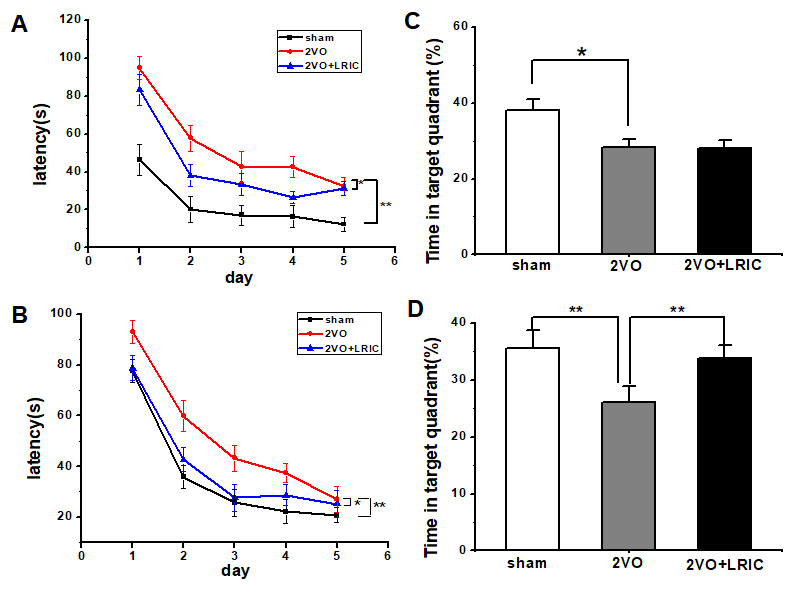
Detection of spatial learning and memory by Morris water maze **A)** Escape latency time by Morris water maze tested from 3 weeks after 2VO surgery. **B)** Percentage of time spent in the target (memory retention ability) at 4 weeks after 2VO surgery. **C)** Escape latency time by Morris water maze tested from 5 weeks after 2VO surgery. **D)** Percentage of time spent (memory retention ability) in the target at 6 weeks. * P<0.05. ** *P*<0.01. N=13/group.

Morris Water Maze test was used to test the effect of LRIC on cognitive impairments after CCH. The escape latency (time required to reach the platform) was used to assess learning capacity. During the learning trials, the rats in all groups showed significant improvement in escape latency time to find the submerged platform (*P*<0.01, repeated-measures ANOVA). The mean escape latency over a period of 5 days for the 2VO group and 2VO+LRIC group was significantly longer than sham group. While the mean escape latency of 2VO+LRIC group was shorter than 2VO group (*P*<?0.05) both for four weeks and six weeks (*P*<0.05, repeated-measures ANOVA; Fig. 2A and C).

The spatial memory ability was assessed by measuring the percentage of time spent in the target quadrant (sham 38.0±2.8%; 2VO 28.4±2.0%; 2VO+LRIC 28.1±2.0% at 4 weeks. sham 35.5±3.2%; 2VO 26.1.5±2.8%; 2VO+LRIC 33.9±2.2% at 6 weeks). Animals in 2VO group spent significantly less time in the target quadrant compared with animals in the sham group at both at 4 and 6 weeks (*P*<0.01, one-way ANOVA. Fig. 2B and D.) There was no significant difference in the percentage of time spent in the target quadrant between the 2VO+LRIC group and the 2VO group at four weeks (*P*>0.05, one-way ANOVA. Fig. 2B). We further continued the LRIC therapy. We found that 2VO+LRIC group spent significantly more time in the target quadrant compared with the 2VO group at 6 weeks (*P*<0.05, one-way ANOVA. Fig. 2D.).

### LRIC prevented cell death in CA1 region after chronic cerebral hypoperfusion

**Figure 3.**
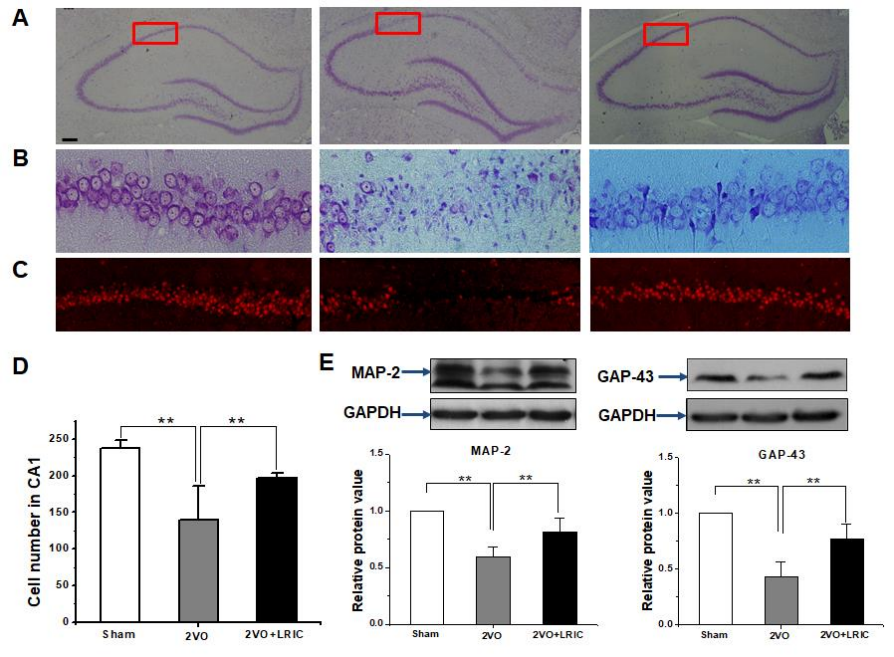
LRIC decreased neuronal cells death after 2VO **A)** Representative images of whole hippocampus stained by Nissl stain. **B)** Images show higher magnifications of CA1 regions as mentioned in A. **C)** Representative images showing expression of NeuN in the CA1 region. **D)** Bar graph shows a quantification of NeuN+ cells. **E)** Western blots assay detected neuronal marker MAP-2 and GAP43 in the hippocampus. ** *P*<0.01,

It is well-known that the hippocampus is involved in spatial learning and memory as assessed by the Morris water maze [3]. We first investigated the pathological change and the expression of NeuN in the hippocampus at week six after 2VO surgery. Cresyl violet staining showed cerebral hypoperfusion induced neuronal cell death and vacuolization in the CA1 region of the hippocampus (Fig. 3A, B). There was a severe loss of NeuN expression in the CA1 region of the 2VO group. LRIC therapy prevented the neuronal cell death and vacuolization in the 2VO+RIPostC group as compared to the 2VO group (Fig. 3C and 3D). Additional studies measured the protein levels of representative markers for neurons, MAP-2 and GAP-43. Western blots showed that LRIC treatment significantly prevented the decrease of MAP-2 and GAP43 (Fig. 3E).

### LRIC treatment increased the vessels number in hippocampus after chronic cerebral hypoperfusion

**Figure 4.**
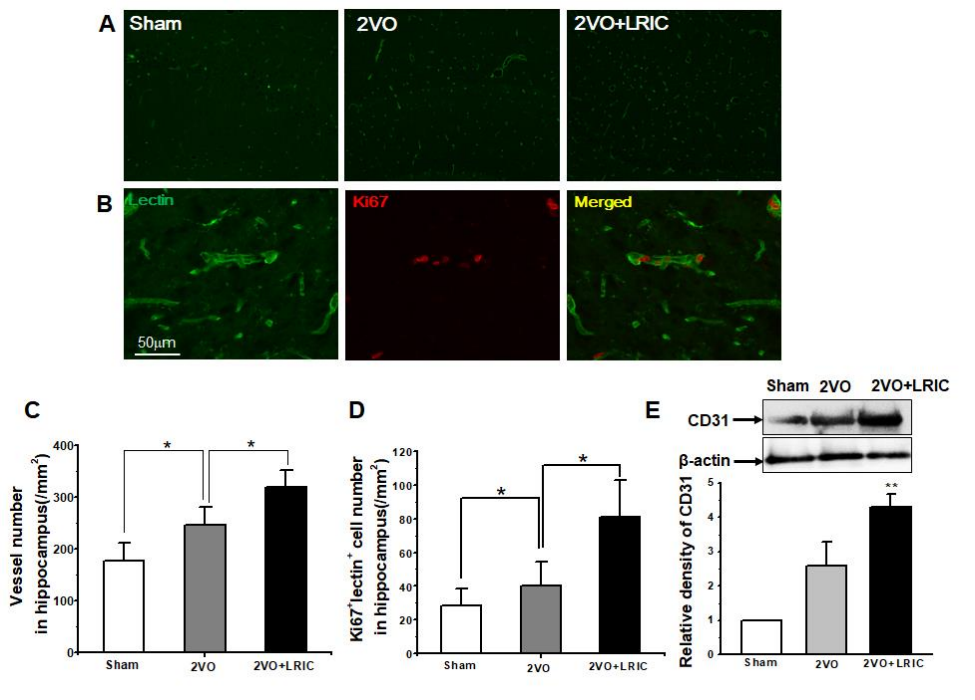
LRIC enhanced the vessels number in the hippocampus after 2VO **A)** Representative images of vessels in the hippocampus detected by lectin. **B)** Images represent double-immunostaining for Lectin (green) and Ki67 (red) cells. **C)** Bar graph shows vessel numbers. **D)** Western blots assay of vessel marker CD31. ** *P*<0.01. N = 5 per group.

Angiogenesis serves as the most efficient mechanism to restore flow after cerebral hypoperfusion. Then we asked whether LRIC treatment can increase the cerebral vessel number in the hippocampus. We performed RECA-1 microvessel immunostaining in the ischemic perifocal region to evaluate the number of microvessels at 28 days following MCAO. The RECA-1+ elements in the hippocampus were significantly increased in 2VO group (246.4±33.0) compared to the sham group (176.4 ± 34.4) (Fig. 4A and 4B; *P*<0.05). LRIC treatment (319.2±33.1) increased the RECA-1+ immunoreactive elements compared to the 2VO group (Fig. 4A and 4B, *P*<0.05). Western blots showed that LRIC increased the protein levels of CD 31, which is representative markers for vascular endothelial cell (Fig. 4C; *P*<0.01).

### LRIC treatment promoted p-eNOS expression after chronic cerebral hypoperfusion

**Figure 5.**
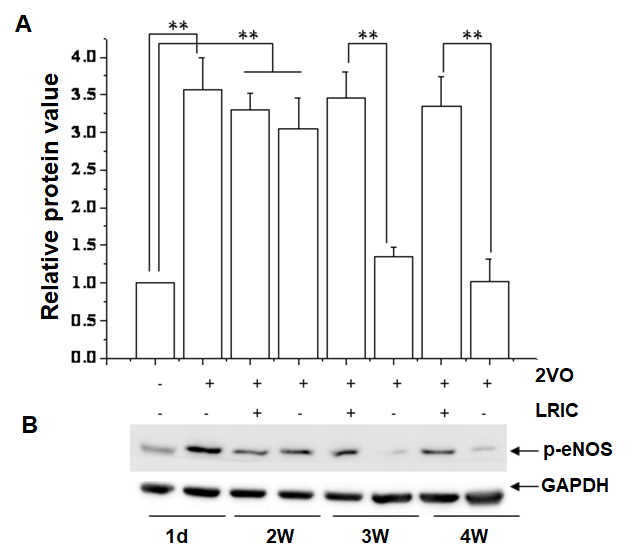
LRIC upregulated the expression of p-eNOS **A)** Bar graph shows a quantification of p-eNOS/GAPDH ratio in different stage after 2VO. ** *P*<0.01. N = 5 per group. **B)** Representative images showing expression of p-eNOS.

The activity of eNOS signaling pathway plays a vital role in ischemic disorders by promoting neovascularization [5]. To explore the possible mechanism of LRIC mediated neuroprotection in 2VO rats, we examined the level of p-eNOS in the hippocampus of CCH rats. Western blot showed that CCH increased the expression of phosphorylated eNOS (p-eNOS) at day 1 (Fig. 5). Further, the high expression of p-eNOS maintained for two weeks. The expression of p-eNOS in 2VO group rat nearly decreased to the baseline level by week three. However, LRIC treatment had a sustained effect on the expression of p-eNOS even up to 4 weeks (Fig. 5)

### NOS inhibitor suppressed the effect of LRIC on angiogenesis and spatial learning and memory and in hippocampus

**Figure 6.**
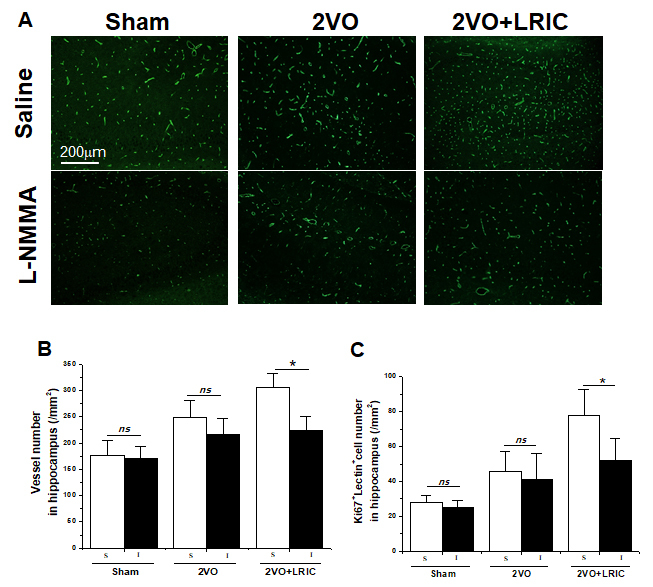
NOS inhibitor suppressed the effect of LRIC on vessels number in hippocampus **A)** Representative images of vessels in the hippocampus detected by lectin. **B)** Bar graph shows a quantification of vessels number. * P<0.05. N = 6 per group. **C)** Bar graph shows a quantification of Lectin^+^/Ki67^+^ cells. N = 6 per group. **P < 0.01.

**Figure 7.**
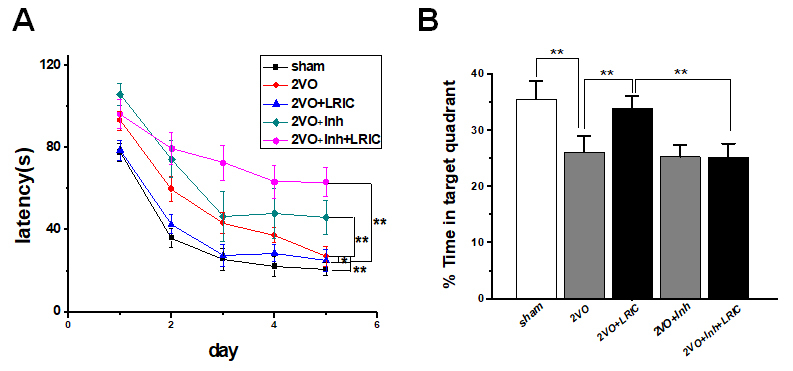
NOS inhibitor suppressed the effect of LRIC on learning and memory behavior **A)** Escape latency time by Morris water maze tested from 5 weeks after 2VO surgery. **B)** Percentage of time spent in the target (memory retention ability) at 6 weeks after 2VO surgery. * P<0.05. ** *P*<0.01. N=13/group.

To determine the role of eNOS/NO signaling in angiogenesis after focal ischemia, NOS inhibitor L-NAME was intraperitoneally injected from day seven after surgery for six weeks. Cerebral vessel number increased after 2VO, which was further elevated by LRIC treatment and reversed by NOS inhibitor treatment (Fig. 6A and 6B). Besides, the number of ki67-positive cells in the cerebral vasculature increased after LRIC treatment when compared to 2VO group, which reversed by NOS inhibitor treatment (Fig. 6C and 6D). The role of NOS in the neuroprotection afforded by LRIC against CCH induced brain injury was further investigated. Spatial learning and memory were detected at week 6 after 2VO surgery. Spatial learning and memory improved with LRIC treatment and was partially blocked by L-NAME (Fig. 6A and 7B).

## DISCUSSION

LRIC treatment significantly increased cerebral perfusion and improved the chronic cerebral hypoperfusion mediated spatial learning and memory impairment compared with the 2VO rats. Immunohistochemistry demonstrated that LRIC treatment prevented cell death in the CA1 region after CCH. Microvessel density and collateral vessels were increased in LRIC-treated rats, suggesting that LRIC promoted local neovascularization in the injured brain. Additional analyses showed that LRIC treatment significantly increased p-eNOS expression in the hippocampus, and NOS inhibitor, L-NAME, suppressed the beneficial effect of LRIC on spatial learning and memory and angiogenesis in the hippocampus. To the best of our knowledge, this is the first study to show that LRIC has a key role in the angiogenesis that follows CCH.

Intracranial and extracranial arterial stenosis is the main cause of CCH. At present, for CCH caused by intracranial and extracranial arterial stenosis, the most effective treatment is the use of carotid endarterectomy, percutaneous vascular angioplasty and stent implantation [19]. However, in the choice of surgery, medical conservatism and other treatment methods have not yet formed a strong clinical evidence. Therefore, for most patients with CCH, in addition to the treatment for risk factors, there is still a lack of effective treatment and drugs [20]. Our previous small size clinical trial study showed that LRIC using a blood pressure cuff increases cerebral perfusion in patients suffering from intracranial arterial stenosis and prevents recurrent stroke [11]. In this study, we found that LRIC treatment significantly increased cerebral perfusion and improved the CCH mediated spatial learning and memory impairment compared with the 2VO rats, which is consistent with the previous study reported by Khan et al. [21].

It is worth mentioning that long-term LRIC therapy can improve both spatial learning and memory impaired. Although LRIC therapy for four weeks did not affect spatial memory, it is interesting that when LRIC therapy was continued up to six weeks, and the percentage of time spent in the target quadrant was measured, LRIC treatment improved impaired spatial memory. Consistent with this study, our previous study demonstrates that LRIC therapy has the long-term feasibility to treat patients for 300 days [13].

Regulation of CBF is critical for the maintenance of neural function. The formation of new blood vessels via angiogenesis is of importance in the restorations of cerebral blood in vascular disease. Thus, manipulation of angiogenesis is an essential clinical goal in vascular disease fields. Our results showed that the number of newly generated vascular endothelial cells labeled with Ki67 and lectin was higher after 2VO, indicating that CCH could induce angiogenesis. In addition, LRIC treatment significantly increased the number of vessels in the hippocampus. A few studies have previously examined the relationship between LRIC treatment and angiogenesis. In agreement with our study, Esposito et al. have found that local ischemic conditioning improves angiogenic remodeling during the recovery phase after focal cerebral ischemia [14]. In addition to these exogenous therapies, exercise is a well-studied therapy that was shown to improve microvascular remodeling, triggering innate mechanisms to improve cognitive impairment after CCH [22]. Limb remote ischemic conditioning, as a noninvasive and no additional cost therapy, can be considered an “exercise equivalent.” Recently, we reported that daily LRIC improved cerebral perfusion in intracranial arterial stenosis patients [13]. With support from the present data, our finding raises the possibility that LRIC may also employ endogenous protective mechanisms to increase angiogenesis.

eNOS/NO pathway is regarded as one of the primary regulators of angiogenesis after ischemia. Activation of eNOS contributes to the maintenance and proliferation of vascular endothelial cells. Vascular remodeling during hind limb ischemia is impaired in eNOS knockout mice [15]. eNOS catalyzes the conversion of L-arginine to L-citrulline generating NO that is important for the angiogenic activity of several factors (e.g., vascular endothelial growth factor, fibroblast growth factor, angiopoietins, platelet-derived growth factors) [5, 23]. And NOS inhibitor L-NAME blunted angiogenesis in a rabbit cornea model [24]. We further observed the effect of LRIC treatment on the expression of p-eNOS. LRIC treatment significantly increased the expression of eNOS compared with the 2VO group at week 3 and 4. Moreover, we also found that the eNOS non-specific inhibitor administration suppressed the effect of LRIC on angiogenesis. Hess et al. reported that LRIC treatment could upregulate the nitrite in plasma, which serves a storage pool of NO derived from endogenous eNOS NO level [25-27]. These results indicated that the eNOS/NO might mediate the effects of LRIC on angiogenesis [28]. To determine whether the inhibition of angiogenesis contributed to neuronal function, we examined spatial learning and memory of rats at week six after 2VO. We found that eNOS non-specific inhibitor administration also suppressed the effect of LRIC on spatial learning and memory after CCH. These results in part suggested that LRIC promoted angiogenesis through the regulation of the expression of eNOS angiogenesis, thus improved cognitive impairment in CCH rats. However, L-NAME is not a specific inhibitor of eNOS, which is the limitation of this study. eNOS knock out mice, or eNOS RNAi approach should be better in this study.

In conclusion, LRIC therapy increased angiogenesis and improveed cognitive function in rat models of CCH. We further demonstrated that LRIC-mediated angiogenesis was reduced in NOS inhibitor administered rats. Our study provided a direct evidence for the involvement of eNOS/NO signaling pathway in angiogenesis in CCH rat models by LRIC.
